# Chronic pancreatitis with pancreatic duct stones complicated by acute obstructive suppurative pancreatic ductitis: Report of a case and review of the literature

**DOI:** 10.1097/MD.0000000000045530

**Published:** 2025-10-31

**Authors:** Xia Ren, Wendi Zhang, Jinbo Wang, Zongyang Wu, Yifeng Wu, Jiye Feng

**Affiliations:** a Department of Hepatobiliary and Pancreatic Surgery, The Affiliated People’s Hospital of Ningbo University, Ningbo, China; b Ningbo University Health Science Center, Ningbo, China.

**Keywords:** acute obstructive suppurative pancreatic ductitis, chronic pancreatitis, endoscopic retrograde cholangiopancreatography, pancreatic duct stones

## Abstract

**Rationale:**

Acute obstructive suppurative pancreatic ductitis (AOSPD) is an exceedingly rare surgical pancreatic condition. This case report aims to elucidate the clinical features and treatment strategies for AOSPD.

**Patient concerns:**

The patient, a 52-year-old male, has a known medical history of chronic pancreatitis, pancreatic duct stones, and type 2 diabetes. In 2022, he received treatment at our hospital through extracorporeal shock wave lithotripsy and endoscopic retrograde cholangiopancreatography. However, in 2023, following a large meal, he experienced persistent upper abdominal pain lasting for 3 days. This episode caused him significant concern about his health, as he began to suspect a potential recurrence of his previous conditions.

**Diagnoses:**

Following the onset of abdominal pain, the patient underwent an emergency endoscopic retrograde cholangiopancreatography at our facility. During the procedure, pus was successfully aspirated from the pancreatic duct for bacterial culture. Subsequent analysis revealed the presence of *Escherichia coli* and *Proteus mirabilis*, which provided conclusive evidence supporting a diagnosis of AOSPD.

**Interventions:**

We promptly relieved the pancreatic duct obstruction in the patients and treated them with antibiotics.

**Outcomes:**

AOSPD primarily affects middle-aged/elderly males, often with chronic pancreatitis, pancreatic duct stones, or pancreatic tumors. Clinical manifestations are nonspecific, with abdominal pain and fever as the most common symptoms. Pancreatic duct obstruction and infection underlie the pathology, making pancreatic duct patency and drainage critical for effective treatment.

**Lessons:**

AOSPD is a rare condition attributed to its low incidence and the challenges associated with diagnosis. AOSPD should be considered in patients with chronic pancreatitis and duct obstruction who present with fever or abdominal pain. Early duct drainage is crucial; however, standardized diagnostic criteria and treatment protocols are still lacking. Further research is warranted.

## 1. Introduction

Acute obstructive suppurative pancreatic ductitis (AOSPD) is an extremely rare clinical entity, with only a handful of documented cases reported in the literature to date.^[1]^ It represents a severe bacterial infection of the pancreatic duct, typically triggered by ductal obstruction or secondary infection associated with pancreatitis.^[2]^ Intestinal bacteria may exhibit retrograde ascent via the duodenal papilla or disseminate through hematogenous pathways, ultimately leading to the accumulation of purulent secretions within the pancreatic duct system. The early symptoms of AOSPD tend to be either nonspecific or relatively mild, often presenting as abdominal pain or nausea. Without timely medical intervention, however, the condition can escalate quickly, potentially leading to severe complications such as septic shock or multi-organ failure. Previously, our hospital managed a case of chronic pancreatitis complicated by pancreatic duct stones and AOSPD, which is summarized in the following report.

## 2. Case report

The patient, a 52-year-old male, was diagnosed in 2022 with chronic pancreatitis, pancreatic duct stones, and type 2 diabetes mellitus due to symptoms of weight loss and abdominal pain. He underwent 2 courses of treatment at our hospital in May and September of the same year, which included extracorporeal shock wave lithotripsy (ESWL) and endoscopic retrograde cholangiopancreatography (ERCP). During the ERCP procedure, stones were removed from the pancreatic duct and an endoscopic retrograde pancreatic drainage stent was placed. The patient has been coping with pancreatic endocrine and exocrine insufficiency through a regimen of insulin injections and enteric-coated pancreatic enzyme supplements. In February 2023, after consuming a particularly heavy meal, he began experiencing persistent upper abdominal pain that lasted for 3 consecutive days. Though the pain was bearable, it was accompanied by noticeable weight loss, diminished appetite, and erratic blood sugar levels that proved difficult to manage. Notably, there were no associated symptoms such as fever, chills, nausea, or vomiting. Given the persistence of these issues, the patient was readmitted to the hospital for further evaluation and targeted treatment.

**Figure 1.**
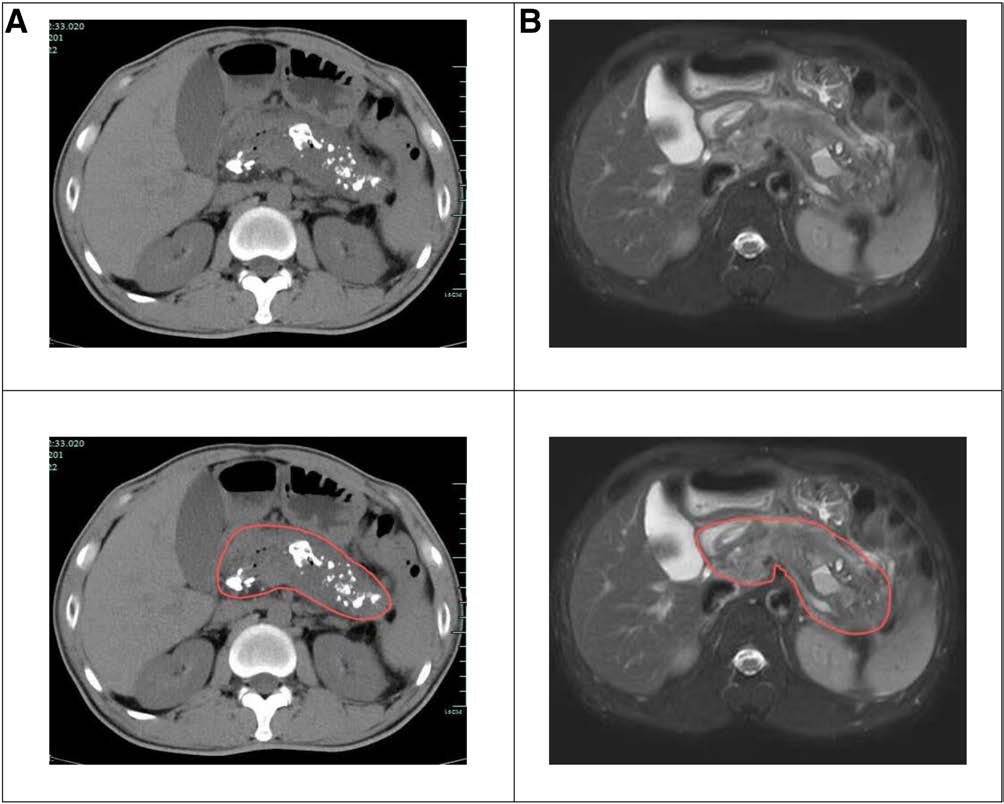
CT and MRI findings of the patient. (A) CT reveals pancreatic swelling with multiple dense foci and minimal gas accumulation. Increased peripheral density is noted, along with mild exudative changes (indicated by the circle). Thickening of the left renal anterior fascia is observed. The pancreatic duct shows bead-like dilation, containing nodular dense shadows and a stent. (B) MRI findings post pancreatic duct stent placement include pancreatic swelling and marked ductal dilation. Multiple small nodular short T2 signal foci are seen within the lumen. The pancreas exhibits increased signal intensity on T2 fat-suppressed imaging, with areas of markedly high signal on DWI. Peripancreatic exudative changes are noted on DWI (indicated by the circle), accompanied by thickening of the left renal anterior fascia.

The physical examination findings upon admission are summarized in Table 1. The patient appeared emaciated but exhibited no scleral or cutaneous jaundice. Subsequent laboratory test results are presented in Table 2. CT and MRI revealed: acute exacerbation of chronic pancreatitis, dilated pancreatic duct with calculi and partial accumulation of pus within the pancreatic duct (Fig. 1).

**Table 1:** Physical examination findings.

Examination item	Result
Body temperature	37.2°C
Blood pressure	118/80 mm Hg
Body weight	50 kg
BMI	19.5 kg/m²
Abdomen	Flat abdomen without visible peristalsis or abdominal wall varicose vein. Tenderness noted in epigastric and mesogastric regions without rebound tenderness or muscle rigidity. No palpable masses or organomegaly
Murphy sign	Negative
Costovertebral tenderness	None
Shifting dullness	Negative
Bowel sounds	Diminished

**Table 2:** Laboratory test results.

Parameter	Result	Reference range
Hematology		
WBC	7.2 × 10^12^/L	3.5–9.5 × 10^9^/L
RBC	4.14 × 10^12^/L	3.8–5.1 × 10^12^/L
Hemoglobin	126 g/L	115–150 g/L
Platelets	247 × 10^9^/L	125–350 × 10^9^/L
Inflammation marker		
CRP	13.73 mg/L	<5 mg/L
Liver function		
ALT	11 U/L	<40 U/L
AST	9 U/L	<35 U/L
Albumin	35 g/L	35–55 g/L
Total bilirubin	7.5 μmol/L	<21 μmol/L
Pancreatic enzymes		
Serum amylase	60 IU/L	30–110 IU/L
Lipid profile		
Total cholesterol	2.93 mmol/L	<5.2 mmol/L
Triglycerides	0.8 mmol/L	<1.7 mmol/L
Electrolytes		
Calcium	2.13 mmol/L	2.1–2.6 mmol/L
Glucose		
Blood glucose	8.59 mmol/L	3.9–6.1 mmol/L
Tumor markers		
CA19-9	7.9 U/mL	<37 U/mL
CA125	16 U/mL	<35 U/mL
CEA	3.46 ng/mL	<5 ng/mL

ALT = alanine aminotransferase, AST = aspartate aminotransferase, CA125 = carbohydrate antigen 125, CA19-9 = carbohydrate antigen 19-9, CEA = carcinoembryonic antigen, CRP = C reactive protein, RBC = red blood cells, WBC = white blood cells.

**Figure 2.**
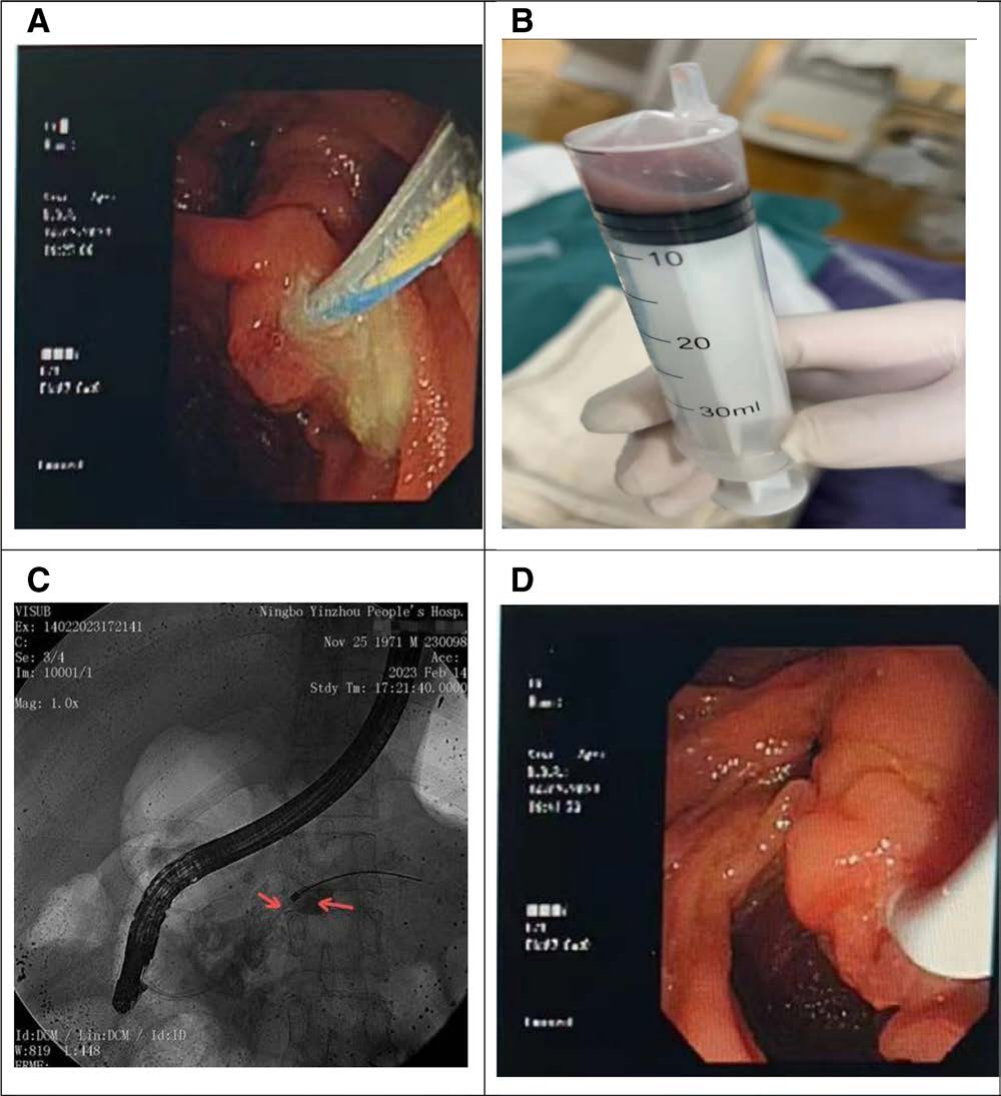
Operations and findings in ERCP. (A) The yellowish-white turbid liquid at the duodenal papilla. (B) 5 mL of purulent fluid with a strong foul odor. (C) X-ray findings: the arrows indicate the location of the stenosis (left) and the dilated pancreatic duct (right). (D) A 7Fr nasopancreatic tube is placed in the body and tail of the pancreas. ERCP = endoscopic retrograde cholangiopancreatography.

On the day of admission, an emergency ERCP was successfully performed. During the procedure, the pancreatic duct stent was carefully removed, followed by dilation of the stricture within the duct. To ensure effective external drainage and decompression, a nasopancreatic drain was strategically placed. After the guidewire and catheter entered the pancreatic duct through the duodenal papilla, a significant amount of yellowish-white turbid fluid was observed flowing out of the duodenal papilla (Fig. 2A), containing white granular particles. Five milliliters of pus were aspirated and sent for bacterial culture (Fig. 2B), which had a noticeable foul odor. Subsequently, a 30% iodine contrast medium was used for pancreatic duct contrast, and X-ray fluoroscopy showed significant dilation of the pancreatic duct in the head of the pancreas, with multiple high-density images within the duct. A significant stricture was observed at the neck of the pancreatic duct, and the duct in the tail of the pancreas was also dilated (Fig. 2C). The guidewire was advanced through the stricture into the body of the pancreas, where the stricture was progressively dilated with 6Fr and 8Fr dilators, ultimately placing a 7Fr nasopancreatic drain. Upon applying negative pressure, yellow-white purulent fluid containing powdery particles was drained from the duct (Fig. 2D).

Postoperatively, the patient’s abdominal pain significantly improved, and bacterial culture results revealed *Escherichia coli* and *Klebsiella oxytoca*, confirming the diagnosis of AOSPD. Antibiotics were adjusted based on the sensitivity test, and the patient’s abdominal pain, blood sugar control, and nutritional status all started to improve. Nine days later, the patient underwent a subsequent ERCP procedure, during which the nasopancreatic drain was replaced with a 7F, 5 cm double-pigtail stent to facilitate internal drainage. The stent was meticulously positioned, with its distal end securely placed in the pancreatic duct at the tail of the pancreas and its proximal end extending into the duodenum, ensuring optimal and uninterrupted drainage. Following the successful treatment, the patient was discharged in stable condition. Over the course of long-term follow-up, 3 additional ERCP procedures were performed for stone extraction. During this period, the patient demonstrated significant improvement—his appetite and body weight progressively increased, blood glucose levels remained stable, and he experienced no recurrence of abdominal pain to date. During the follow-up period, the patient reported a marked improvement in symptoms, accompanied by a restoration of sound mental well-being. In light of the rarity of this condition, we took the opportunity to discuss the potential value of publishing a case report. The patient, upon receiving a comprehensive explanation, not only provided informed consent but also expressed enthusiastic and full support for this initiative.

## 3. Discussion

AOSPD is a relatively rare condition first reported in 1995,^[3]^ However, its pathogenesis and diagnostic criteria are still subject to debate.^[4]^ Current evidence indicates that the development of AOSPD is closely linked to pancreatic duct obstruction, increased intraductal pressure, and retrograde infection. Through an analysis of various studies (Table 3),^[2–16]^ we discovered that the majority of AOSPD patients are middle-aged and elderly males.^[17]^ These patients commonly present a medical history marked by chronic pancreatitis, pancreatic duct stones, pancreatic tumors, or prior ampullary surgery. Their clinical manifestations are notably nonspecific, primarily characterized by abdominal pain and fever. Although less frequent, cases of shock and sepsis can also occur, adding complexity to the clinical picture. Pancreatic exocrine and endocrine functions typically remain unaffected in most clinical presentations. Laboratory findings predominantly reveal leukocytosis, while serum amylase levels and liver function parameters generally stay within normal ranges in the majority of cases. Imaging examinations reveal pancreatic duct dilation with localized strictures, often accompanied by pancreatic duct stones and pancreatic calcification, which are features of chronic pancreatitis, without obvious acute pancreatic exudation or peripancreatic edema. Pain relief is usually achieved by pancreatic duct drainage (either endoscopic or surgical). Intraoperative findings include purulent pancreatic juice and positive bacterial cultures. In this case, the patient had chronic pancreatitis and pancreatic duct stones. Abdominal pain occurred due to post-ESWL stone fragments obstructing the pancreatic duct. CT and MRI showed pancreatic swelling with pus accumulation in the duct lumen. ERCP confirmed pancreatic duct obstruction and infection. The rapid improvement of symptoms following nasopancreatic drainage of purulent pancreatic fluid provides compelling evidence that AOSPD is closely associated with pancreatic duct obstruction, elevated ductal pressure, and retrograde infection.

**Table 3:** Summary of previous AOSPD case reports.

Case	Age	Sex	Etiology	Interventions	Outcomes
Hirata^[5]^	61	Male	Calculi within the main pancreatic duct of the pancreatic head	ERCP	The inflammation improved, but pyogenic spondylitis occurred
Nakayama^[6]^	77	Female	Intraductal papillary mucinous neoplasms	Endoscopic nasopancreatic drainage (ENPD)	Swelling of the pancreatic tail was completely improved
Oura^[7]^	63	Male	A pancreatic duct stricture in the pancreatic head and dilatation of the caudal pancreatic duct	ERCP	The patient was discharged, without clinical symptoms
Kitagawa^[8]^	72	Male	*Raoultella planticola*	Endoscopic drainage of pancreatic ducts and antibiotic therapy	The patient’s symptoms have been relieved
Pallaneeandee^[9]^	40	Female	An inflamed head of the pancreas, with a stone, and duodenal obstruction	Pancreaticoduodenectomy	The patient’s symptoms were relieved, and she recovered fully
Shimizuguchi^[10]^	64	Male	Pancreatic ductal adenocarcinomas	ERCP	The patient’s symptoms have been relieved
Shimizuguchi^[11]^	80	Female	Pancreatic cancer	Nasopancreatic drainage (NPD)	The patient’s condition and laboratory result improved
Iwatsuka^[12]^	68	Male	Pancreatic cancer	ENPD and pancreaticoduodenectomy	The patient’s symptoms did not worsen after being controlled
Inoue^[13]^	70	Male	Type 1 autoimmune pancreatitis	ERCP	The patient’s symptoms and laboratory test data have significantly improved
Tollivoro^[14]^	56	Female	Pancreatic duct stones	ERCP and antibiotic treatment	The patient’s symptoms have been relieved
Isono^[15]^	85	Female	Pancreatic head cancer	ERCP and ENPD	The patient’s symptoms have been relieved
Wali^[4]^	63	Male	*Escherichia coli, Streptococcus pneumoniae*, and *Haemophilus influenzae*	ERCP	The patient has never had any clinical symptoms
Nishie^[16]^	60	Male	Intraductal papillary mucinous carcinoma (IPMC)	ENPD	The patient’s symptoms have been relieved
Fujinaga^[2]^	70	Male	Branch duct intraductal papillary mucinous neoplasm and pancreatolithiasis	ERCP, ENPD, and antibiotic treatment	The patient recovered quickly with no fever and the abdominal pain
Fujimori^[3]^	53	Male	Dilation of the main pancreatic duct and impaction of pancreatic stones	Endoscopic nasobiliary drainage (ENBD)	The patient’s symptoms have improved significantly
Our case	52	Male	Pancreatic duct stones	ERCP and antibiotic treatment	The patient’s abdominal pain symptoms were significantly improved, and both blood glucose control and nutritional status were improved

AOSPD = acute obstructive suppurative pancreatic ductitis, ERCP = endoscopic retrograde cholangiopancreatography.

The primary pathological mechanism of AOSPD is pancreatic duct obstruction and compromised immunity.^[2]^ Normal pancreatic juice is alkaline and contains a wealth of antibacterial digestive enzymes, which play a crucial role in maintaining intestinal mucosal integrity. Typically, common bacteria do not proliferate within the pancreatic duct. However, in the event of obstruction, the consequent rise in intraductal pressure, stasis of pancreatic juice, and ductal dilation not only precipitate significant abdominal pain but also compromise the antibacterial activity of the pancreatic fluid. If a retrograde bacterial infection breaches the immune defenses within the pancreatic duct, obstructive suppurative pancreatic ductitis may ensue. Our review of multiple case reports has revealed several common obstructive factors (Table 4).^[6,12,18,19]^ Nayagan et al^[9]^ reported a case of AOSPD secondary to duodenal obstruction, which was successfully managed through surgical relief of the obstruction, further highlighting the central role of pancreatic duct obstruction in AOSPD pathogenesis. Furthermore, epidemiological evidence suggests that several conditions associated with immunocompromise—including long-term steroid therapy, diabetes mellitus, peripheral blood stem cell suppression, and systemic lupus erythematosus–can significantly facilitate both the proliferation and translocation of enteric pathogens. Smoking and alcohol consumption may,^[20]^ independently or synergistically, accelerate the progression of chronic pancreatitis, induce changes in pancreatic parenchyma, and impair the intestinal mucosal barrier function, thus further increasing susceptibility to infections. Hirata et al reported a case of AOSPD complicated by pancreatic abscess that developed pyogenic spondylitis despite endoscopic drainage,^[5]^ highlighting the critical role of host immunity in disease progression.

**Table 4:** Summary of obstructive causes in various case reports.

Case	Age	Sex	Cause of obstruction	Pathological mechanism
Gedam^[18]^	33	Female	Chronic pancreatitis	Long-standing inflammation reduces antimicrobial activity and flow rate, predisposing to bacterial infection
Iwatsuka^[12]^	68	Male	Pancreatic neoplasms	Tumor-induced ductal compression combined with immunocompromised status promotes infection
Wang^[19]^	76	Male	Endoscopic interventions	ERCP-related procedures (e.g., guidewire insertion, sphincterotomy) may introduce enteric bacteria
Nakayama^[6]^	77	Female	Diabetes and intraductal papillary mucinous neoplasms	Mucin plugs obstruct the pancreatic duct, and underlying conditions such as diabetes mellitus further compromise immunity, increasing susceptibility to infection
Our case	52	Male	Pancreatic duct stones	Calculi induce strictures and distal dilation, exacerbating infection risk

ERCP = endoscopic retrograde cholangiopancreatography.

Retrograde bacterial infection, translocation, and virulence also constitute significant pathogenic factors in the development of diseases. In the microbiology of AOSPD, Enterobacteriaceae (especially *Klebsiella* and *Escherichia coli*) are predominant,^[6]^ although *Citrobacter freundii* and *Enterobacter cloacae* are occasionally isolated as well. Importantly, Shou Kitagawa et al reported a case associated with *Raoultella planticola*,^[8]^ which suggests a wider diversity of pathogens.

Maintaining the patency and effective drainage of the pancreatic duct is crucial for the successful treatment of AOSPD. In the diagnosis of AOSPD—characterized by symptoms such as abdominal pain and fever, a documented history of chronic pancreatitis, imaging evidence indicating pancreatic duct obstruction, and infection-related laboratory abnormalities—it is imperative to promptly initiate decompression of the pancreatic duct alongside empirical antibiotic therapy. Subsequent adjustments to the antibiotic regimen can then be made according to the results of pancreatic juice cultures. ERCP-guided pancreatic duct drainage stands out as the preferred treatment due to its minimally invasive nature and repeatability, demonstrating efficacy in 95% of AOSPD cases.^[10]^ When paired with extracorporeal shock wave lithotripsy (ESWL) for stone fragmentation, this method not only effectively relieves pancreatic duct obstruction but also significantly diminishes the recurrence rates. For patients facing challenges with ERCP cannulation, altered upper gastrointestinal anatomy, or concurrent pancreatic masses, EUS-guided pancreatography and drainage may theoretically offer superior diagnostic and therapeutic outcomes compared to ERCP.^[21]^ In cases where endoscopic treatment fails to yield satisfactory results, pancreatic duct stones remain inadequately controlled, or a pancreatic tumor is suspected, surgical intervention to alleviate obstruction should be carefully considered. Some experts argue that ERCP stent drainage might result in insufficient drainage due to complications such as stent occlusion, migration, or retrograde infection. While more invasive procedures like pancreaticoduodenectomy may be necessary, they can effectively address both pancreatic duct and duodenal obstructions while enabling pathological diagnosis.^[9]^ Gedam et al^[18]^ reported a case where AOSPD was successfully treated with “pancreatic duct exploration with stone extraction plus pancreaticojejunostomy.” This surgical approach not only effectively tackled the root cause of the obstruction, ensuring optimal ductal drainage, but also eradicated the necessity for repeated endoscopic interventions. Consequently, it brought about a marked improvement in both the patient’s symptoms and overall prognosis. In our case, the patient’s medical history played a pivotal role in guiding the surgical team to promptly consider biliary-pancreatic pathology as the source of abdominal pain. Emergency ERCP confirmed AOSPD, and nasopancreatic drainage provided substantial relief. Following the acute phase, internal pancreatic duct stenting was performed to maintain duct patency, facilitating subsequent ESWL and ERCP-guided stone removal. This minimally invasive strategy yielded excellent results, markedly alleviating pancreatic duct obstruction.

Early symptoms of AOSPD are typically mild or insidious, often resulting from a confluence of factors. In instances of incomplete pancreatic duct obstruction, partial drainage of pancreatic juice alleviates intraductal pressure and restricts infection spread, thereby attenuating symptom severity. Temporary relief caused by mobile calculi or mucin plugs can lead to fluctuating or minimal clinical signs, further complicating early detection. Variations in immune competence among patients—such as those with diabetes, advanced age, or immunocompromised status—can dampen inflammatory responses, contributing to diverse symptom presentations. In our patient, preexisting chronic pancreatitis likely induced adaptive tolerance, explaining the subdued abdominal pain despite acute pathological changes. As clinicians, we must remain vigilant to avoid underestimating the condition based on its initially mild symptoms. A thorough review of medical history and imaging studies is critical for timely diagnosis. Once diagnosed, immediate intervention is imperative. Beyond symptomatic management, definitive treatment strategies should include antimicrobial therapy and measures to relieve pancreatic duct obstruction—these not only address acute pathology but also mitigate the risk of progression to severe complications.

## 4. Conclusion

AOSPD is clinically rare because of its low incidence and frequent underdiagnosis. Patients with chronic pancreatitis or pancreatic duct obstruction who present with fever and abdominal pain should be assessed for AOSPD. Although early ductal drainage is crucial, standardized diagnostic criteria and treatment protocols are still lacking, highlighting the need for further pathological research.

## Author contributions

**Funding acquisition:** Jiye Feng.

**Investigation:** Jinbo Wang, Zongyang Wu.

**Methodology:** Jinbo Wang, Zongyang Wu.

**Supervision:** Yifeng Wu, Jiye Feng.

**Validation:** Zongyang Wu.

**Writing—review & editing:** Xia Ren, Wendi Zhang, Jiye Feng.

**Writing—original draft:** Wendi Zhang.

## References

[1] Tajima Y Kuroki T Susumu S. Acute suppuration of the pancreatic duct associated with pancreatic ductal obstruction due to pancreas carcinoma. Pancreas. 2006;33:195–7. 16868487 10.1097/01.mpa.0000226891.71075.4c

[2] Fujinaga T Nishida T Miyazaki M. Acute suppurative pancreatic ductitis associated with pancreatic duct obstruction. Endoscopy. 2013;45:E135. 23716098 10.1055/s-0032-1326450

[3] Fujimori N Igarashi H Ito T. Acute obstructive suppurative pancreatic ductitis. Clin Gastroenterol Hepatol. 2011;9:A28. 10.1016/j.cgh.2011.03.012 21421076

[4] Wali E Koo P Packer CD. Acute obstructive suppurative pancreatic ductitis in an asymptomatic patient. Case Rep Med. 2015;2015:919452. 25688269 10.1155/2015/919452 PMC4320860

[5] Hirata A Matsumori T Yasuda M. A rare case of acute obstructive suppurative pancreatic ductitis (AOSPD) which developed pyogenic spondylitis. Clin J Gastroenterol. 2024;17:982–8. 38902593 10.1007/s12328-024-02004-y

[6] Nakayama S Fukuda A Nishikawa S. A case of spontaneous acute obstructive suppurative pancreatic ductitis associated with intraductal papillary mucinous neoplasms. Clin J Gastroenterol. 2024;17:760–4. 38709443 10.1007/s12328-024-01973-4

[7] Oura H Sugiyama H Nishino T. A case of acute obstructive suppurative pancreatic ductitis complicated with acute cholangitis diagnosed only after the removal of a pancreatic duct stent. DEN Open. 2024;4:e352. 38515612 10.1002/deo2.352 PMC10956771

[8] Kitagawa S Tsushima Y Ishikawa S. Acute obstructive suppurative pancreatic ductitis caused by raoultella planticola: an emerging pathogen. Pancreas. 2022;51:e118–9. 37099795 10.1097/MPA.0000000000002158

[9] Pallaneeandee NK Govindan SS Liu ZJ. Acute obstructive suppurative pancreatic ductitis (AOSPD) with duodenal obstruction treated by pancreaticoduodenectomy (PD): a rare case report. J Int Med Res. 2022;50:3000605221133152. 36369720 10.1177/03000605221133152 PMC9676295

[10] Shimizuguchi R Kikuyama M Kamisawa T Kuruma S Chiba K. Acute obstructive suppurative pancreatic ductitis in pancreatic malignancies. Endosc Int Open. 2020;8:E1765–8. 33269309 10.1055/a-1268-7086 PMC7671758

[11] Shimizuguchi R Kikuyama M Kamisawa T Kuruma S Chiba K. Acute obstructive suppurative pancreatic ductitis (AOSPD) in pancreatic cancer treated by nasopancreatic drainage. Clin J Gastroenterol. 2018;11:315–9. 29464657 10.1007/s12328-018-0830-z

[12] Iwatsuka K Nakagawara H Ogawa M. Spontaneous development of acute obstructive suppurative pancreatic ductitis associated with pancreatic carcinoma: a first case report. Intern Med. 2018;57:1241–5. 29279516 10.2169/internalmedicine.9862-17 PMC5980803

[13] Inoue T Ito K Ishii N Kobayashi Y Yoneda M. Acute obstructive suppurative pancreatic ductitis associated with type 1 autoimmune pancreatitis. Pancreas. 2017;46:e24–5. 28187113 10.1097/MPA.0000000000000794

[14] Tollivoro T Palakodeti S Munroe C. Acute obstructive suppurative pancreatic ductitis. ACG Case Rep J. 2016;3:e136. 27807588 10.14309/crj.2016.109 PMC5062672

[15] Isono Y Matsusaki S Tanaka H. Acute obstructive suppurative pancreatic ductitis after endoscopic retrograde cholangiopancreatography in a patient with carcinoma of the pancreatic head:a case report. Nihon Shokakibyo Gakkai Zasshi. 2016;113:289–95. 26853989 10.11405/nisshoshi.113.289

[16] Nishie H Okumura F Fukusada S. A case of intraductal papillary mucinous carcinoma found with acute obstructive suppurative pancreatic ductitis and liver abscess, and associated with a pancreatobiliary fistula. Nihon Shokakibyo Gakkai Zasshi. 2013;110:1304–12. 23831662

[17] Kondo H Naitoh I Okumura F. Clinical features of acute obstructive suppurative pancreatic ductitis: a retrospective review of 20 cases. J Gastroenterol Hepatol. 2016;31:1366–73. 26840231 10.1111/jgh.13304

[18] Gedam BS Sadriwala QS Bansod PY. Chronic pancreatitis with acute obstructive suppurative pancreatic ductitis: a rare case report. J Surg Case Rep. 2017;2017:rjx034. 28458839 10.1093/jscr/rjx034 PMC5400493

[19] Wang Z Zhang PP Bai Y Wang D. A rare case of acute obstructive suppurative pancreatic ductitis associated with ERCP. Rev Esp Enferm Dig. 2019;111:73. 30284907 10.17235/reed.2018.5756/2018

[20] Hansen SEJ Nordestgaard BG Langsted A. Smoking as the most important risk factor for chronic pancreatitis in the general population. Eur J Epidemiol. 2023;38:95–107. 36593333 10.1007/s10654-022-00945-7

[21] van der Merwe SW van Wanrooij RLJ Bronswijk M. Therapeutic endoscopic ultrasound: European society of gastrointestinal endoscopy (ESGE) guideline. Endoscopy. 2022;54:185–205. 34937098 10.1055/a-1717-1391

